# Randomized, Double-Blinded, Double-Dummy, Active-Controlled, and Multiple-Dose Clinical Study Comparing the Efficacy and Safety of Mulberry Twig (Ramulus Mori, Sangzhi) Alkaloid Tablet and Acarbose in Individuals with Type 2 Diabetes Mellitus

**DOI:** 10.1155/2016/7121356

**Published:** 2016-07-28

**Authors:** Mengyi Li, Xuemin Huang, Hui Ye, Yao Chen, Jing Yu, Jinxia Yang, Xuezhi Zhang

**Affiliations:** ^1^Department of Integrated Traditional Chinese Medicine and Western Medicine, Peking University First Hospital, Beijing 100034, China; ^2^Department of Integrated Traditional Chinese Medicine and Western Medicine, Dongzhimen Hospital, Beijing University of Chinese Medicine, Beijing 100007, China

## Abstract

*Aims*. To evaluate the efficacy and safety of mulberry twig alkaloid (SZ-A) tablet compared with acarbose in patients with type 2 diabetes.* Methods*. This clinical trial enrolled 38 patients who were randomized into two groups (SZ-A: 23; acarbose: 15) and were treated for 24 weeks. Patients and clinical trial staffs were masked to treatment assignment throughout the study. The primary outcome measures were glycated hemoglobin (HbA1c) and 1-hour and 2-hour postprandial and fasting plasma glucose levels from baseline to the end of treatment. Analysis included all patients who completed this study.* Results*. By the end of this study, HbA1c level in SZ-A group was decreased from baseline significantly (*P* < 0.001). No significant difference was found when compared with acarbose group (*P* = 0.652). Similarly, 1-hour and 2-hour postprandial plasma glucose levels in SZ-A group were decreased from baseline statistically (*P* < 0.05), without any significant differences compared with acarbose group (*P* = 0.748 and 0.558, resp.). The fasting plasma glucose levels were not significantly changed in both groups. One of 23 patients in SZ-A group (4.76%) and 5 of 15 patients in acarbose group (33.33%) suffered from gastrointestinal adverse events.* Conclusions*. Compared with acarbose, SZ-A tablet was effective and safe in glycemic control in patients with type 2 diabetes.

## 1. Introduction

Diabetes is one of the largest global health emergencies of the 21st century. In most countries, diabetes and its complications are the leading causes of death. The scary number of diabetes has been increasing in most regions. In 2015, 415 million adults are estimated to currently have diabetes and 318 million adults with impaired glucose tolerance in the world [1]. Moreover, due to the cost of essential medicines, diabetes has a substantial economic impact on individuals, their families, and national health systems [1]. In addition to public health education and early diagnosis, effective treatment of diabetes is the indispensable role in halting the rise of diabetes.

Among three main types of diabetes, type 2 diabetes is the most prevalent form. Patients with early type 2 diabetes may be able to maintain normal blood glucose levels by means of a meal plan and physical exercise. As the disease progresses, oral hypoglycemic drugs are indicated. To decrease the postprandial rise in glucose levels in these patients, carbohydrate absorption can be decreased or delayed by the prandial use of acarbose, an *α*-glucosidase inhibitor that acts on the small intestine by blocking the digestion of complex carbohydrates [2]. However, the most frequent gastrointestinal side effects of acarbose are flatulence and diarrhea, due to its mechanism of action [3]. In a STOP-NIDDM randomized trial, the percentage of gastrointestinal adverse events of acarbose was 13% (flatulence 9%, diarrhea 5%, abdominal pain 3%, and other 1%), which were more frequent than in those given placebo (*P* < 0.0001) [4].

Herbal medicine has been wildly used to treat type 2 diabetes in Asia for centuries. Many antidiabetic herbs are adopted extensively in clinical units with proven efficacy and safety [5–8]. Moreover, screening of *α*-glucosidase inhibitors from plants has been a hot research topic [9–11]. The most reported plant is mulberry (Latin name:* Morus alba* L.) [12–16], one kind of herbs, which is commonly used in Chinese medicine. Mulberry twig (Latin name:* Ramulus Mori*, Chinese name: Sang Zhi, SZ), is the dry branch of mulberry, which is widely distributed in Asia.

1-Deoxynojirimycin (1-DNJ) is a main active constituent of effective fraction of alkaloids from SZ [12]. It has been reported that 1-DNJ is an *α*-glucosidase inhibitor [17–19] which can delay glucose absorption and significantly reduce postprandial blood glucose levels [20]. In a previous study [21], the effective fraction of alkaloids from SZ (SZ-A) was found to have a strong *α*-glucosidase inhibitory activity* in vitro* and* in vivo*. Compared with acarbose, SZ-A showed stronger inhibition of sucrase (IC_50_ = 21.9 ng/mL), equal inhibition of maltase (IC_50_ = 40.4 ng/mL), and less inhibition of amylase* in vitro* [22]. Also, in the study of blood glucose of normal mice after loading sucrose, the results showed that the SZ-A in the dosage of 10 mg/kg–40 mg/kg can significantly reduce elevated blood glucose, and the blood glucose area under the curve was significantly less than acarbose group. The results of these two groups (10 mg/kg–40 mg/kg) had no significant differences. In another study of blood glucose of alloxan induced diabetic mice after loading sucrose, the results showed that the SZ-A could lower and postpone the peak of blood glucose and reduce the blood glucose area under the curve. The effects of 20 mg/kg and 40 mg/kg SZ-A treatment groups were better than acarbose groups. These results suggested that the hypoglycemic activity of SZ-A was similar to acarbose [21, 22].

In our study, we compared the efficacy and safety of SZ-A tablet with acarbose for 24 weeks. Monotherapy and polytherapy with metformin were also included. The aims of this study were to (1) evaluate the efficacy and safety of SZ-A tablet, (2) find the lowest effective dose of SZ-A tablet (as the same effectiveness of acarbose), and (3) investigate whether the polytherapy with metformin has better effects on glycemic control, compared with monotherapy. This is the first clinical study of SZ-A tablet compared with acarbose in patients with type 2 diabetes.

## 2. Materials and Methods

### 2.1. Patients and Study Design

Eligible patients in the study were 18–70 years of age, with a diagnosis of type 2 diabetes according to the 1999 World Health Organization diagnostic criteria, who were not on a regimen of antidiabetic medical treatment at least 3 months before screening, who were on a regimen of antidiabetic treatment no more than 3 months at any time in the past, who were on a stable regimen of metformin monotherapy for at least 8 weeks, and who had a glycated hemoglobin concentration (HbA1c) ≥7.0% (53 mmol/mol) and ≤10.0% (86 mmol/mol), a fasting plasma glucose level ≤13 mmol/L (234 mg/dL), and a body mass index (BMI) of 19–30 kg/m^2^. Patients were excluded for a difference of fasting plasma glucose levels between 1st follow-up and 2nd follow-up >2.5 mmol/L (45 mg/dL), severe diabetes complications (e.g., diabetic ketoacidosis), allergy to or intolerance of *α*-glucosidase inhibitors, confounding concomitant drug use (including insulin, incretin mimetics, thiazolidinediones, antidiabetic herbal medicine, and glucocorticoids), poor blood pressure control (SBP > 160 mmHg or DBP > 100 mmHg), liver disease, kidney disease, intestinal conditions (e.g., inflammatory bowel disease), substantial alcohol consumption (>20 g/day for women or >30 g/day for men), pregnancy, and disorders such as a medical history of major pathology. Written informed consent was obtained from all patients before the trial began (to convert mmol/L to mg/dL, multiply by 18).

This was a randomized, double-blinded, double-dummy, active-controlled, and multiple-dose clinical trial evaluating the efficacy and safety of SZ-A tablet compared with acarbose in patients with type 2 diabetes. Eligible patients were randomly assign by a computer-generated, centrally administered randomization schedule via an interactive web response system (IWRS). Each patient was associated with a randomization code. Allocation concealment was achieved by packaging both SZ-A and acarbose groups with a unique identification number by the manufacturer. Patients, investigators, clinical trial staffs, and physicians were masked to treatment assignment throughout the study. Emergency unblinding service is provided. The double-dummy design was accomplished by use of the placebo formulations of SZ-A and acarbose. In order to be indistinguishable in terms of odor and taste, SZ-A placebo contains 1/20 the dosage of SZ-A, which is an ineffective dose, while acarbose placebo imitates acarbose tablet in appearance and weight.

The tablets SZ-A (each tablet 50 mg) were produced by Beijing Wuhe Boao Pharmaceutical Technology Development Co., Ltd., which was approved to produce tablets in September 2008 by CFDA (China Food and Drug Administration, number 2008L05752). An effective fraction of alkaloids of SZ-A is prepared from mulberry twig and the active ingredients are a composition of alkaloids, including N-methyl-1-deoxynojirimycin (1-DNJ), 3-epi-fagomine, fagomine, 1,4-dideoxy-1,4-imino-D-arabinitol, and 1,4-dideoxy-1,4 imino-(2-O-*β*-D-glucopyranosyl)-D-arabinitol. Among these alkaloids, 1-DNJ is the highest content [21]. The tablets acarbose (each tablet 50 mg) were produced by Bayer Healthcare Pharmaceutical Inc., Germany. In January 22, 2014, the Ethics Committees of Peking University First Hospital where the study was conducted approved the trial protocol. The study was registered at http://db.yaozh.com/ (CTR20140034).

To improve accuracy and screen compliance, all enrolled patients underwent a 4-week lead-in period (1st follow-up: −4 weeks), given SZ-A placebo tablet 50 mg and acarbose placebo tablet 50 mg with first bite of food, three times daily (t.i.d.). After randomization, all patients underwent a multiple-dose procedure: for SZ-A group, its initial dose is SZ-A tablet 50 mg t.i.d. + acarbose placebo tablet 50 mg t.i.d (for 4 weeks) and maximum dose is SZ-A tablet 100 mg t.i.d. + acarbose placebo tablet 50 mg t.i.d. (till end of treatment); for acarbose group, its initial dose is acarbose tablet 50 mg t.i.d. + SZ-A placebo tablet 50 mg t.i.d. (for 4 weeks) and maximum dose is acarbose tablet 50 mg t.i.d. + SZ-A placebo tablet 100 mg t.i.d. (till end of treatment).

### 2.2. Study Endpoints and Assessments

Patients returned for study visits at weeks −4, 0, 4, 8, 16, and 24 (end of treatment). Patients had physical examination, routine blood tests, urine tests, and electrocardiogram (ECG) during each visit. The primary objective of the study was to demonstrate effectiveness of SZ-A tablet on HbA1c change during the period, compared with acarbose. Secondary objectives were to evaluate the changes of fasting plasma glucose level and 1-hour and 2-hour postprandial plasma glucose levels during the study.

Safety assessments included adverse events, hypoglycemia, vital signs (blood pressure), ECGs, and laboratory variables. Hypoglycemia was defined as a measured plasma glucose concentration ≤3.9 mmol/L and/or symptoms and/or signs attributable to hypoglycemia [23]. Severe hypoglycemia was defined as an episode requiring the assistance of another person to actively administer therapy.

Patients were not given any additional therapy during the trial. No dose reductions of SZ-A, acarbose, or their placebo were allowed throughout the 24-week treatment period. Participants with previous treatment of metformin were continued at the same dose as before randomization.

### 2.3. Statistical Analysis

We used SPSS 17.0 software (SPSS, Inc., Chicago, IL) for all statistical analysis. For normally distributed quantitative data, independent-samples *t*-test or one-way analysis of variance (ANOVA) was used to test the effects of treatment on the changes of HbA1c, plasma glucose levels, and other routine blood tests; paired *t*-test was used to test the effects of each group before and after the treatment. If the drug effect was found to be significant by ANOVA, multiple comparison of LSD was used to test the difference between the different treatments. Qualitative data were analyzed by using Chi-square (Fisher's exact test or Monte Carlo exact test) or nonparametric technique using Kruskal-Wallis test. All tests were two-tailed, and the level of significance was set at 0.05 (*P* < 0.05).

## 3. Results

### 3.1. Patients

**Figure 1:**
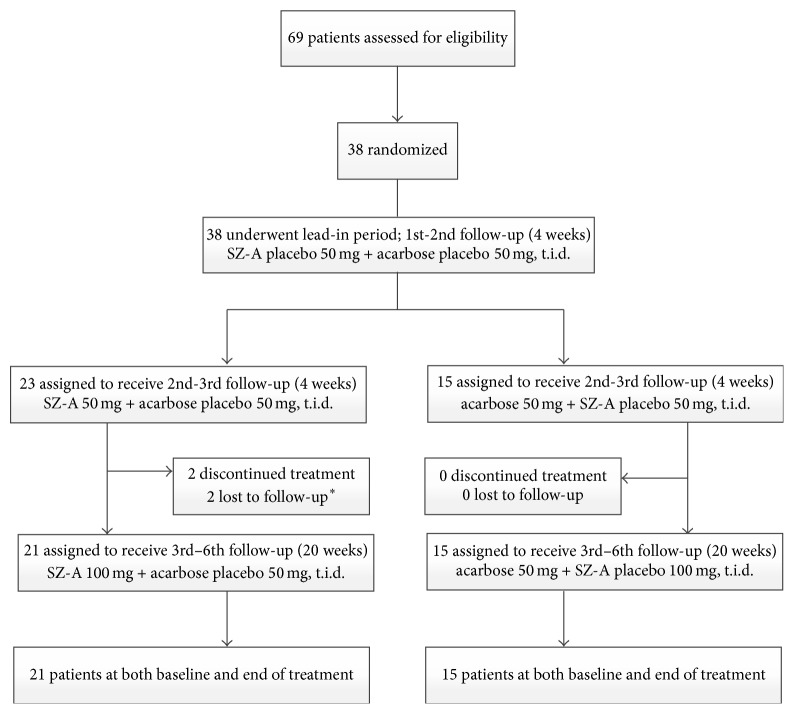
Trial profile. ^*∗*^ Two patients assigned to the SZ-A group were lost to follow-up (one patient was lost at week 4 after receiving drugs; one patient was lost at week 8 after receiving drugs) because of the failure to getting in contact with them.

From June 25, 2014, to December 29, 2014, 69 patients were recruited in this study. After systemic review, we excluded 31 patients who met our exclusive criteria. Finally, 38 patients were randomly assigned to receive SZ-A (*n* = 23) or acarbose (*n* = 15). Two patients in SZ-A group were lost to follow-up because of the failure of getting in contact with them. No patient was lost to follow-up in acarbose group (Figure 1). We analyzed the records of 36 patients in total, 21 patients in SZ-A group, and 15 patients in acarbose group, respectively. Baseline demographic, clinical, and laboratory features were similar in the two groups (Table 1).

### 3.2. Efficacy

Twenty-one (91%) patients in SZ-A group and 15 (100%) patients in acarbose group had group-comparison and self-comparison in terms of HbA1c, fasting plasma glucose levels, postprandial plasma glucose levels, lipids, liver functions, and kidney functions both at baseline and at week 24 (Table 2).

**Figure 2:**
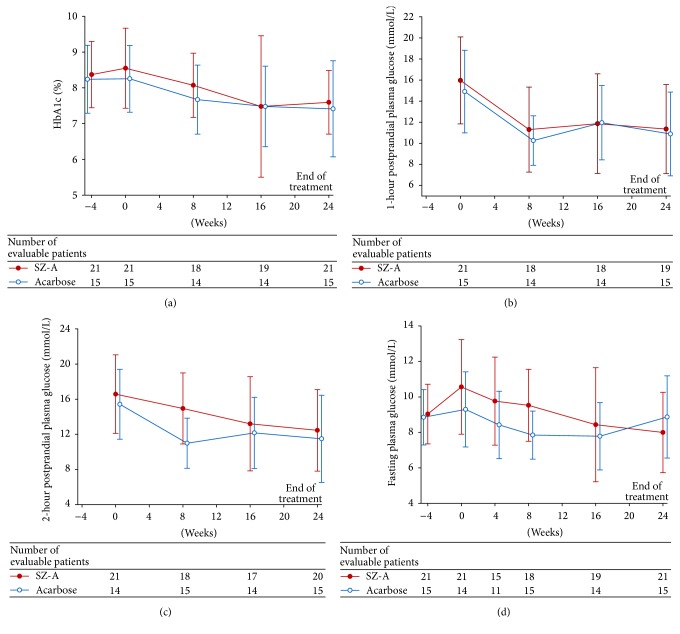
Glycated hemoglobin (HbA1c), postprandial plasma glucose, and fasting plasma glucose from baseline to week 24. (a) The difference of HbA1c between SZ-A group and acarbose group was not statistically significant (*P* > 0.05). (b) The difference of 1-hour postprandial plasma glucose levels was not significant in group-comparison (*P* > 0.05). (c) The difference of 2-hour postprandial plasma glucose levels was not significant in group-comparison (*P* > 0.05). (d) The difference of fasting plasma glucose levels was not significant in group-comparison (*P* > 0.05).

For the self-comparison in each group, the significant changes from baseline to 24 weeks in HbA1c were −0.776% (*P* < 0.001) and −0.827% (*P* < 0.05) (SZ-A and acarbose group, resp.). Compared with acarbose, SZ-A reduced HbA1 with 95% confidence interval (CI): 0.18 (−0.64 to 1.00). The differences between two groups were not statistically significant (Figure 2(a); *P* > 0.05). As for postprandial plasma glucose levels, treatments in both groups significantly decreased 1-hour postprandial plasma glucose levels from baseline to 24 weeks (self-comparison; Table 2; *P* < 0.05), while the difference between groups was not significant (Figure 2(b); *P* > 0.05). Similarly, in each group, 2-hour postprandial plasma glucose levels were significantly decreased from baseline to 24 weeks (self-comparison; Table 2; *P* < 0.05), while the difference in group-comparison was not significant (Figure 2(c); *P* > 0.05). Both treatments did not change fasting plasma glucose levels significantly in self-comparison and group-comparison (Figure 2(d); *P* > 0.05).

There was no significant difference in lipids, liver functions, or kidney functions between the two groups from baseline to end of treatment (Table 2).

**Figure 3:**
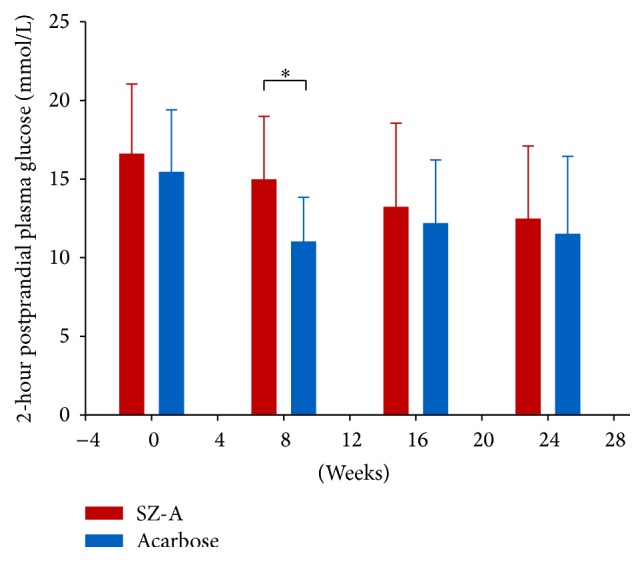
Two-hour postprandial plasma glucose levels in group-comparison from baseline to week 24. It showed a significant reduction in patients receiving acarbose at week 8 compared with SZ-A (^*∗*^ *P* < 0.05).

In addition, the results of 2-hour postprandial plasma glucose levels in group-comparison showed a significant reduction in patients receiving acarbose at week 8 compared with SZ-A (Figure 3; *P* < 0.05).

**Figure 4:**
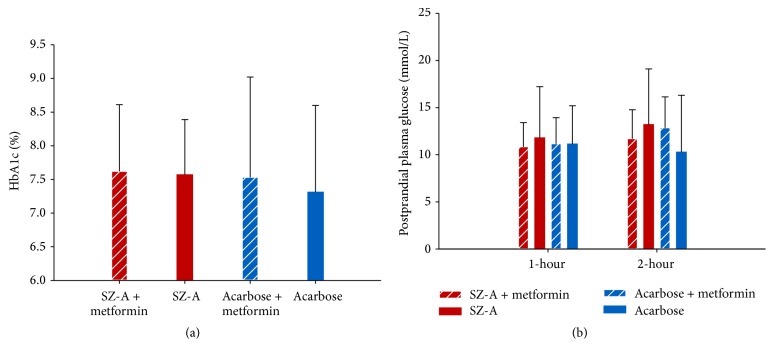
Comparisons of HbA1c and postprandial plasma glucose levels between monotherapy and polytherapy at week 24. (a) The difference of HbA1c among SZ-A + metformin, SZ-A, acarbose + metformin, and acarbose groups was not significant at week 24. (b) The differences of 1-hour and 2-hour postprandial plasma glucose levels between four groups were not significant at week 24.

In comparison of monotherapy and polytherapy with metformin, there was no significant difference between four treatment groups (SZ-A + metformin, SZ-A, acarbose + metformin, and acarbose groups) in terms of HbA1c (*P* = 0.945), fasting plasma glucose (*P* = 0.720), and 1-hour and 2-hour postprandial plasma glucose levels (*P* = 0.940, *P* = 0.597, resp.; Figures 4(a)-4(b)).

### 3.3. Safety

Adverse events were recorded in detail in the two treatment groups in each follow-up (Table 3). The two patients in the SZ-A group withdrew from treatment because of failure to contact them. There were no serious adverse events (deaths, heart failure, liver failure, or kidney failure and other conditions) during the treatment period. All gastrointestinal adverse events were considered to be of mild intensity. The percentage of gastrointestinal disorders in SZ-A group was obviously lower than that of in acarbose group, which included increased release of gas (one (4.76%) patients in SZ-A group versus 2 (13.33%) in acarbose group) and diarrhea (none versus 3 (20%)).

According to liver and kidney function tests recorded at each visit, the level of increased alanine aminotransferase, aspartate aminotransferase, and urine acid and the level of reduced creatinine clearance rate in SZ-A group were slightly higher than that in acarbose group. Long-time clinical observation is needed and we should pay more attention to the cause-effect relationship between SZ-A tablet and mildly increased aminotransferase and urine acid or reduced creatinine clearance rate. We noticed that the abnormal creatinine clearance rate of two patients in SZ-A group and two patients in acarbose group turned to be normal after the study. We also noted that there were two (9.52%) urinary tract infection cases in the SZ-A group, both of which were considered unrelated to the treatments. Changes from baseline in ECG are shown in Table 3. No cardiovascular events were observed.

## 4. Discussion

Intensive glucose control is the predominant factor to prevent the development of chronic complications in type 2 diabetes [24–27]. An important component of dysglycemia is postprandial hyperglycemia [28]. Postprandial hyperglycemia remains a prominent feature in the early stage of diabetes and has been demonstrated in Chinese patients with type 2 diabetes [29]. Sustained postprandial hyperglycemia is an independent risk factor for cardiovascular complications and death [30]. There were two main categories of the treatment of postprandial hyperglycaemia: (1) therapeutic agents that act on intestinal digestion of carbohydrates and (2) therapeutic agents that stimulate or mimic the postprandial insulin response [27]. The *α*-glucosidase inhibitor belongs to the first category and plays a significant role in pharmacological treatments to control postprandial glycemic excursions. Acarbose was the first agent of *α*-glucosidase inhibitor to be made available and adopted extensively.

In this randomized, double-blinded, double-dummy, active-controlled clinical trial, the *α*-glucosidase inhibitor, SZ-A, met the predefined primary endpoint and led to significant reduction of HbA1c and postprandial plasma glucose in 24-week treatment period. Fasting plasma glucose levels had no significant reduction in both groups (SZ-A and acarbose group), which indicated that fasting blood glucose is poorly sensitive to *α*-glucosidase inhibitors. In addition, there was no statistical difference of HbA1c levels in both SZ-A and acarbose group. Similar results were presented in terms of postprandial plasma glucose levels in group-comparison. That is, compared with acarbose 50 mg t.i.d., the dose of SZ-A tablet 100 mg t.i.d. can achieve similar hypoglycemic effects.

According to a previous unpublished randomized, dose-escalation, active-controlled, multicenter phase II clinical study, SZ-A tablets 100 mg (once taken) showed the similar effects as acarbose 50 mg (once taken) on reducing postprandial blood glucose levels by inhibiting and delaying digestion and absorption of carbohydrate. In this study, from the beginning to the end of week 4, we applied SZ-A 50 mg t.i.d.; after week 4, we applied a dose-escalation of SZ-A tablet to 100 mg t.i.d., while the dose of acarbose still remained 50 mg t.i.d. As a result, compared with SZ-A group, the 2-hour postprandial plasma glucose level was significantly decreased (*P* = 0.003) in the patients in acarbose group only at week 8 but was not shown in other follow-up weeks or records. Similar to the previous clinical study, we could not acquire an ideal hypoglycemic effect on 2-hour postprandial plasma glucose level when SZ-A tablet 50 mg t.i.d. was applied. This result was reflected by SZ-A group at week 8; even the dose-escalation of SZ-A tablet had been applied for 4 weeks (from week 5 to week 8) in this study. This could be explained by the fact that drugs always need a time period to reach a stable drug concentration and reveal its best efficacy. Dose-escalation for only 4 weeks was not quite enough for SZ-A tablet. However, we continued dose-escalation to 100 mg t.i.d. of SZ-A till end of treatment, and there was no significant difference in 2-hour postprandial plasma glucose after 8 weeks. Besides, in terms of HbA1c and 1-hour postprandial plasma glucose levels, no significant difference appeared in group-comparison during the whole 24 weeks, whether the dose-escalation of SZ-A applied or not. We considered that 50 mg t.i.d. SZ-A tablet had similar hypoglycemic effects on HbA1c and 1-hour postprandial plasma glucose as the same dose of acarbose. In the consideration of the drawback of SZ-A on 2-hour postprandial plasma glucose control, 100 mg t.i.d. of SZ-A should be recommended in clinical practice for patients with type 2 diabetes mellitus as monotherapy. Further studies are still needed to investigate whether a combination of low dose of SZ-A (e.g., 50 mg t.i.d.) with other hypoglycemic treatments could acquire better efficacy of 2-hour postprandial plasma glucose control.

Combination therapy is commonly prescribed in the treatment of type 2 diabetes. The combination of acarbose and metformin is recommended in clinic because of their different and complimentary mechanisms of action, which provides effective glycemic control with additional cardiovascular benefits and minimizes adverse events [31, 32]. In this study, the combination therapy of SZ-A and metformin was also applied without any adverse events. But there was no significant difference in primary outcome measures between monotherapy and polytherapy with metformin. Further large-scale clinical study with long-time follow-ups should be conducted to assess the effects of combination of SZ-A and metformin on glycemic control and provide more clinical evidence.

*α*-Glucosidases are membrane-bound enzymes that digest disaccharides such as amylase, maltose, and sucrose in the small intestine [33]. Gastrointestinal side effects are one of the limitations of *α*-glucosidase inhibitor in clinical application. Our study also demonstrated this kind of side effect in both SZ-A and acarbose groups. However, the percentage of gastrointestinal side effects in patients receiving SZ-A was lower than that in acarbose group. The reason for this result should be carefully discussed. Acarbose, an aminooligosaccharide isolated from the fermentation broth of* Actinoplanes sp.*, inhibits brush-border *α*-glucosidases in humans [34, 35]. Acarbose can give rise to major adverse effects such as abdominal distention, flatulence, meteorism, and possibly diarrhea [4]. Such adverse effects are attributed to the impaired digestion of starch by strong inhibition of intestinal *α*-amylase. When undigested starch increases, it could be hydrolyzed by the bacteria residing in the colon and be used for fermentation, releasing gas and low-molecular-weight substances [36]. Then, this abnormal fermentation results in several undesirable side effects as mentioned above [36]. Compared with acarbose, plant-derived *α*-glucosidase inhibitor, mulberry leaf extract (MLE) has lower *α*-amylase inhibitory activity. The* in vitro* inhibitory activity of MLE on intestinal *α*-glucosidase was potent and that on intestinal *α*-amylase was very weak compared with acarbose [37]. Similar results were also reported in previous* in vitro* experiment of SZ-A [21]. Thus, SZ-A tablet is a strong *α*-glucosidase inhibitor but has less *α*-amylase inhibitory activity. SZ-A could be effective for postprandial hyperglycemia with minimal side effects. To some extent, it will improve the patient's compliance.

Limitations of our study should be considered when interpreting the results. Firstly, sample size was not large. Comparative study about effects of SZ-A and acarbose on glycemic control in patients with type 2 diabetes has not yet been published. Thus, the estimate of sample size was based on the feasibility of conducting a clinical trial. Albeit similar to other proof-of-concept studies [5, 38], our sample size was smaller than some later stage studies [39]. Despite this, every patient was strictly matched for features of type 2 diabetes and inclusion criteria in our study. Secondly, observation time was only 24 weeks. Due to the previous phase II clinical study, it was shown that the gastrointestinal side effects of SZ-A mainly occurred within 4 weeks after receiving tablets. With the extension of received time, the better tolerance for SZ-A was observed. For other adverse events, we think that our study should be with an extension (such as 28 weeks mentioned in a previous study [39], or maybe even longer observation period [40]). Thirdly, we did not analyze the BMI changes in our study. We collected the baseline BMI data (weight and height) of eligible patients when screening for the first time, but we missed some data in follow-ups. Further investigations with larger sample size and longer observation time are still needed to clarify the safety and efficacy of SZ-A tablet.

In conclusion, the efficacy and safety of SZ-A (mulberry twig alkaloid) tablet in reducing postprandial plasma glucose levels and HbA1c render it an attractive therapy for individuals with type 2 diabetes.

## Figures and Tables

**Table 1:** Baseline characteristics of trial population.

	SZ-A (*n* = 23)	Acarbose (*n* = 15)	*P* value
Demographics			
Age (years)	56 (9.71)	57 (6.70)	0.747^a^
Gender (male/female)	8/15	5/10	1.000^b^

Comorbidities			
Hypertension	6 (26.1%)	6 (40.0%)	0.481^b^
Hyperlipidaemia	17 (73.9%)	10 (66.7%)	0.722^b^

Concomitant drug use			
Metformin	12 (52%)	7 (47%)	1.000^b^
Antilipidemic	2 (0.09%)	0	NA
Antihypertensive	1 (0.04%)	0	NA

Metabolic factors			
Disease duration (months)	36 (6,180)	30 (9,120)	0.800^c^
Fasting glucose (mmol/L)	8.94 (1.63)	8.85 (1.56)	0.873^a^
HbA1c (%)	8.30 (0.91)	8.24 (0.95)	0.847^a^
Weight (kg)	71.63 (12.06)	69.30 (13.00)	0.576^a^
BMI (kg/m^2^)	25.67 (2.744)	25.47 (2.612)	0.822^a^
Systolic blood pressure (mmHg)	131.48 (11.735)	129.67 (15.267)	0.682^a^
Diastolic blood pressure (mmHg)	81.39 (8.217)	78.67 (9.123)	0.345^a^
Smoking history (current smoker)	1 (4.3%)	4 (26.7%)	0.069^b^
Drinking history (current drinker)	2 (8.7%)	0	NA
Proteinuria			0.898^c^
0	12 (63.2%)	9 (64.3%)	—
Trace	5 (26.3%)	4 (28.6%)	—
1+	2 (10.5%)	1 (7.1%)	—
Glycosuria			
0	12 (63.2%)	7 (50.0%)	1.000^b^
Trace	2 (10.5%)	3 (21.4%)	0.365^d^
1+	0	1 (7.1%)	NA
2+	0	0	NA
3+	2 (10.5%)	3 (21.4%)	0.365^d^
4+	3 (15.8%)	0	NA

Lipids			
Total cholesterol (mmol/L)	5.17 (1.06)	4.85 (0.91)	0.350^a^
Triglycerides (mmol/L)	2.26 (1.30)	1.89 (1.12)	0.367^a^
HDL (mmol/L)	1.18 (0.37)	1.15 (0.27)	0.803^a^
LDL (mmol/L)	3.04 (0.93)	2.97 (0.69)	0.790^a^

Liver function tests			
Alanine aminotransferase (U/L)	22.39 (9.41)	26.60 (15.04)	0.294^a^
Aspartate aminotransferase (U/L)	17.91 (5.43)	20.07 (6.27)	0.269^a^

Kidney function tests			
Creatinine clearance (mL/min)	77.08 (20.66)	76.59 (14.56)	0.937^a^
Uric acid (mmol/L)	292.91 (80.62)	241.20 (90.95)	0.078^a^

ECG			
Normal	11 (57.89%)	7 (50%)	1.000^b^
ST segment depression	2 (10.53%)	0 (0%)	NA
T wave nonspecific changes	3 (15.79%)	3 (21.43%)	0.663^d^
Low voltage	1 (5.26%)	0 (0%)	NA
High voltage of left ventricle	1 (5.26%)	1 (7.14%)	1.000^d^
PR interval shortened	1 (5.26%)	0 (0%)	NA
Left ventricle hypertrophy	0 (0%)	1 (7.14%)	NA
Right atrium hypertrophy	0 (0%)	1 (7.14%)	NA
Sinus bradycardia	0 (0%)	1 (7.14%)	NA

Compliance (%)	97.66 (2.95)	97.36 (2.08)	0.734^a^

Data are mean (SD) or *n* (%) or median (minimum, maximum). ^a^Independent-samples *t*-test. ^b^Chi-square tests (Fisher's exact test). ^c^Nonparametric tests. ^d^Chi-square tests (Monte Carlo exact test).

**Table 2:** Changes in plasma glucose levels, lipids, liver functions, and kidney functions from baseline to week 24.

	Mean (SD) change from baseline to week 24				Mean (95% CI) changes from baseline (SZ-A versus acarbose)	*P* value
	SZ-A (*n* = 21)	*P* value	Acarbose (*n* = 15)	*P* value		
Plasma glucose levels						
Fasting glucose (mmol/L)	1.04 (2.61)	0.083	−0.02 (2.25)	0.976	−0.88 (−2.45 to 0.70)	0.266
Postprandial blood glucose 1 h (mmol/L)	4.10 (5.11)	0.003^*∗*^	4.02 (4.92)	0.007^*∗*^	0.46 (−2.44 to 3.36)	0.748
Postprandial blood glucose 2 h (mmol/L)	3.84 (5.05)	0.003^*∗*^	3.83 (5.83)	0.029^*∗*^	0.97 (−2.36 to 4.29)	0.558
HbA1c (%)	0.78 (0.85)	0.000^*∗*^	0.83 (1.35)	0.033^*∗*^	0.18 (−0.64 to 1.00)	0.652

Lipids						
Total cholesterol (mmol/L)	0.06 (1.21)	0.824	−0.01 (0.65)	0.969	0.30 (−0.36 to 0.96)	0.366
Triglycerides (mmol/L)	0.10 (1.17)	0.706	0.31 (0.93)	0.214	0.61 (−0.11 to 1.33)	0.094
HDL (mmol/L)	−0.04 (0.19)	0.386	−0.02 (0.15)	0.527	0.07 (−0.14 to 0.27)	0.513
LDL (mmol/L)	−0.09 (0.72)	0.586	0.06 (0.49)	0.634	0.25 (−0.21 to 0.71)	0.271

Liver functions						
Alanine aminotransferase (U/L)	−0.38 (8.39)	0.837	2.07 (14.21)	0.582	−2.53 (−9.77 to 4.70)	0.482
Aspartate aminotransferase (U/L)	0.33 (4.20)	0.720	1.27 (6.15)	0.438	−2.28 (−5.50 to 0.95)	0.160

Kidney functions						
Creatinine clearance (mL/min)	2.36 (13.94)	0.447	−2.56 (16.30)	0.553	−3.71 (−17.02 to 9.61)	0.575
Urine acid (mmol/L)	−5.55 (58.59)	0.760	−12.83 (38.79)	0.455	36.50 (−40.03 to 113.03)	0.327

^*∗*^ *P* < 0.05.

**Table 3:** Adverse events.

	SZ-A (*n* = 23)	Acarbose (*n* = 15)
Overall treatment withdrawal rate	2 (0.09%)	0

Treatment withdrawal due to adverse event	0	0

Participants with serious adverse event	0	0

Adverse event		
Gastrointestinal disorders	1 (4.76%)	5 (33.33%)
Increased release of gas	1 (4.76%)	2 (13.33%)
Diarrhea	0	3 (20%)
Abdominal pain	0	0
Dyspepsia	0	0
Flatulence	0	0
Urinary tract infection	2 (9.52%)	0
Increased alanine aminotransferase (mild)	2 (9.52%)^*∗*^	0
Increased aspartate aminotransferase (mild)	1 (4.76%)^*∗∗*^	0
Reduced creatinine clearance (mild)	1 (4.76%)	0
Increased urine acid	1 (4.76%)^*∗∗∗*^	0
ECG changes from normal		
Sinus bradycardia	1 (4.76%)	0
T wave nonspecific changes	1 (4.76%)	0
ST segment ischemic changes	1 (4.76%)	0

No deaths were reported in the trial period (weeks 0–24). ^*∗*^One patient's alanine aminotransferase was normal on further testing until end of treatment. ^*∗∗*^This patient's aspartate aminotransferase was normal on further testing until end of treatment. ^*∗∗∗*^This patient was lost to follow-up.
